# Measuring socioeconomic and health financing inequality in maternal mortality in Colombia: a mixed methods approach

**DOI:** 10.1186/s12939-020-01219-y

**Published:** 2020-07-31

**Authors:** Juan Carlos Rivillas, Raúl Devia-Rodriguez, Marie-Gloriose Ingabire

**Affiliations:** 1 Maternal and Child Health Program (MCH), International Development Research Centre (IDRC), Ottawa, Canada; 2 School of Medicine, University of Valle, Cali, Colombia

**Keywords:** Health financing, Maternal health, Inequality, Health insurance, Equity, Universal health coverage

## Abstract

**Background:**

Understanding health financing reforms and means is key to evaluate how maternal health has improved. Problems related to health financing policies are contributing to inadequate quality of care and inequitable use of healthcare by pregnant women, resulting in poor maternal health outcomes. The purpose of the study was to measure socioeconomic and health financing related inequality in maternal mortality in Colombia as well as identifying potential epicenters of this inequality.

**Methods:**

The data used was obtained from National Information of Social Protection (Sispro), the Department of Planning and National Statistics Department. Maternal mortality ratios were calculated by health insurance scheme and disaggregated by health spending per capita quintiles to allow for closer examination of inequality. The Slope Index of Inequality and Concentration Index were estimated to express absolute and relative inequality. We conducted interviews with key informants involved in the implementation of health financing and maternal health policies.

**Results:**

The main finding shows inequality in maternal mortality across regions and in particular in the subsidized health insurance. The contributory health insurance scheme is closing gaps over time, but inequality in the subsidized scheme is significantly widening, which impacts the severity of overall measurements of inequality. 20% of territories with the lowest health spending per capita have reached 35% of maternal mortality, and it such rates are worsening. This means that there is a marginal exclusion in which most of maternal deaths still occur in the regions with lowest resources.

**Conclusions:**

Beyond the key issues in health financing, issues of quality of care must be addressed. The country must define its own approach to financing for maternal health coverage given its unique situation and starting point. Potential policy implications that emerged are: i) afro-Colombian, indigenous, poorer and migrant women must be put at the center of the maternal health care services; ii) better skills, Reproductive, Maternal, Newborn and Child Health RMNCH training and health worker retention strategies and training in rural, insular and remote geographical areas; ii) a better understanding of provider payment mechanisms and the incentives that influence provider behaviors; and iv) inequality prompt calls for a targeted approach, whereby care is directed toward the most disadvantaged regions.

## Background

The innovation of the sustainable development goals (SDGs) is that they are interconnected, comprehensive and multi-sectoral targets. Thus, achieving specific goals depends on the progress in achieving other goals. As part of the SDGs, health is captured explicitly under the SDG-3 aimed at *“Ensuring healthy lives and promoting well-being for all at all ages”.* This goal includes reducing the global maternal mortality ratio to less than 70 per 100,000 live births to achieve universal health coverage by 2030. Multi-country studies [1, 2] show that universal health coverage is a critical component of sustainable development, and a key element in reducing social inequity and poverty, in particular in health financing reforms and policies.

The definition of Universal Health Coverage (UHC) embodies three specific policy goals: i) equity in the use of health services; ii) quality of care; and iii) universal financial protection. While no country in the world has fully achieved these three “UHC goals”, each has sought to make progress in these categories; hence, moving toward universal health coverage requires progress on the three UHC goals that are embedded in the definition [3]. Despite the commitment by all countries to move towards universal health coverage, countries are still struggling to design and implement adequate approaches. Experts [4, 5] acknowledge that even under the most favorable planning scenarios, many countries still face tremendous financial and organizational constraints in attaining universal health coverage. Health financing arrangements influence progress on these UHC goals directly, but also through their effects on three intermediate objectives with implications for UHC: equity in distribution, transparency and accountability; and efficiency [6]. New resources and tools have been developed to provide guidance in addressing health financing constraints [3]. This guidance insists on the importance of a solid descriptive overview of the health financing system as a basis for identifying areas that are causing underperformance of the system relative to the ultimate and intermediate objectives of the universal health coverage. To achieve this, countries must pay attention to each of the three health financing functions: i) raising funds in an equitable way, ii) pooling to spread risk protection for all and iii) effective mechanisms to purchase health services.

Inadequate health financing in health systems is contributing to inequitable access to quality health services on the basis of need (e.g. family planning, antenatal care visits, newborn care, etc.). In turn, this underperforming system leads to poor maternal health outcomes. There is a close link between maternal health and health financing arrangements and functions. The Maternal Mortality Ratio (MMR) is one of the common indicators of the quality of health care and is used for comparative analysis of health systems and as a general measure of population health and wellbeing over time [7–9].

Despite the mortality ratio dropping by 44% worldwide from 1990 to 2015, 99% of maternal deaths are still happening due to the lack of access to skilled routine and emergency care. In addition, the highest concentration of these maternal deaths occur in low and middle income countries [10]. Maternal survival has significantly improved since the adoption of the MDG-5 in Latin America and in Caribbean countries, along many other regions: the maternal mortality ratio has nearly been reduced by half and most of the reduction has occurred since 2000. However the region is still far from the 2015 target [7, 11]. Thus, significant efforts will be necessary in order to meet the sustainable development goals.

Colombia has seen a significant reduction in maternal mortality. Mortality decreased by 34% over a fifteen-year period, however the country did not meet the MDG-goal (45 maternal deaths per 100,000 live births); in 2015, it reported 64 maternal deaths per 100,000 live births. Yepes [12] argues that this is unacceptable for a country that has introduced heath care reforms from 1993 with the purpose to improve institutional delivery care, antenatal care and financial protection during maternal health [13, 14].

However, while at the country-level mortality decreased, the inequality in mortality varied significantly at the subnational level. The latest health analysis [15] shows how departments such as Choco and La Guajira –two regions characterized as the most rural and dispersed areas, represent the two regions with considerably high mortality ratios: 224.61 and 135.81 per 100,000 live births respectively. Moreover, they have the highest concentration of maternal deaths among afro-Colombian and indigenous girls and women.

Before 1993, Colombia had a public health model (National Health system) under a centralized health system, which covered around 40% of the population. After 1993, the General Social Health Security System established a model of a comprehensive insurance scheme based on health market competition between public and private health care providers, and financing functions were transformed in order to achieve a more equitable distribution and efficient management of resources. As a result, three health insurance schemes were introduced: i) Contributory, i.e. people with the resources to pay; ii) Subsidized; people under the scheme of solidarity; and iii) Exception, a special regime for people that belong to special groups such as military forces, teachers, and certain government entities. The majority of the population is covered by the first two schemes (46% contributory and 45% subsidized health insurance schemes). The subsidized regime is a mandatory scheme for the unemployed, informal sector workers and the poor (as determined by a means test), including their dependents. Members of this regime do not make contributions to the regime, which is financed in a complex fashion through different sources, mainly general taxes [13].

However, in 2012, there was another significant health reform in Colombia with the universalization of packages of health services under the two major schemes. For example, before 2012, a pregnant woman under the subsidized scheme would have to pay for hospitalizations, lab tests and family planning, which were covered under the contributory scheme. However, after 2012, the services of these two regimes were combined. Ruiz and Zapata [16] and Guerrero and colleagues [13] suggest that health financing in Colombia is progressive.

As we described above, Colombia shows an inequitable progress within the country. Our hypothesis is that health financing reforms are contributing to achieving the universal health coverage goals (equity in the use of health services, quality of care and universal financial protection) and as a result, influencing inequality in maternal mortality. The goal of this research was twofold: first, to explain the patterns of inequality in maternal mortality driven by health financing. Second, to understand the key factors of health financing reforms related to revenue collection, pooling and purchasing health services, all which affect the implementation of the maternal health policy.

## Methods

### Study design

**Fig. 1:**
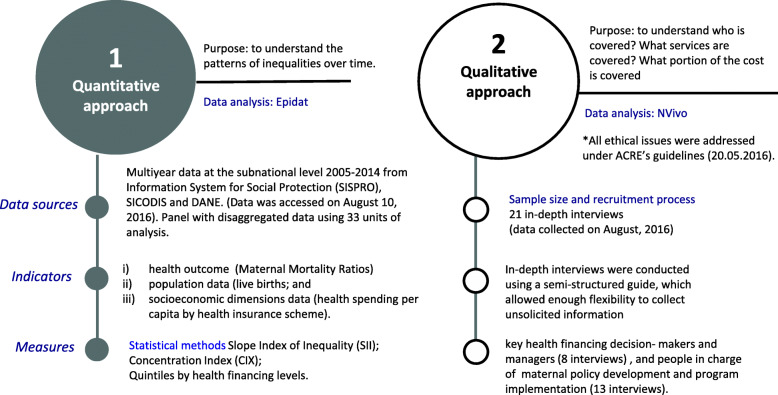
Research methodology approach and purpose. Source: prepared by authors from study results

This was an ecological study at the subnational level and used a mixed methods approach which combines quantitative and qualitative research methodologies. Figure 1 shows a flow diagram to explain the study’s methods along with the purposes for these methods.

### Settings and data collection

We used country-data disaggregation at the sub-national level (33 geographical units). All data was collected during July and August 2016. Firstly, the quantitative research approach used a cross-sectional study and a secondary multi-year disaggregated panel data linking three different sources of data: i) health outcomes (maternal mortality); ii) population (live births); and iii) socioeconomic dimensions (health spending).

Maternal Mortality Ratios (2005–2014) were obtained from the Cube of mortality registries sourced by Information System for Social Protection (SISPRO) and compared with Indicadores Basicos en Salud (IBS), both available from the Ministry of Health and Social Protection of Colombia [17]. This Cube is the result of a consensus that consolidates information on deaths and was validated with Vital Statistics from the National Administrative Department of Statistics (DANE) [18]. Health spending per capita data was obtained from the Office for Regulation of Benefits and Insurance (Contributive health insurance Scheme 2009–2014), and from Information System of Regional Distributions of Resources (SICODIS) (Subsidized health insurance Scheme 2007–2014) [19]. The health spending per capita data was collected from these two sources, but data were only comparable for the 2012–2014 period. Data on live births was provided by Department of Epidemiology and Demography of the Ministry of Health and Social Protection for the period between 2005 and 2014, which contains annual information of demography at sub-national levels from DANE. This data was exported and saved in a combined panel with disaggregated data using 33 units of analysis (which correspond to the 32 departments and Bogota, D.C), each one by health insurance scheme (contributory and subsidized) for the years 2012–2014. Detailed information about the four available databases and their corresponding time series is described in the Table 1. All data is publicly available and can be accessed at www.sispro.gov.co.

**Table 1:** Absolute and relative inequality in maternal mortality by health spending per capita and maternal mortality ratios disaggregated data by health spending per capita quintiles, 2009 and 2014

Health financing indicators	Slope inequality of index (SII)		Concentration index (CIX)	Q1 (lowest)	Q2	Q3	Q4	Q5 (Highest)
	Absolute inequality	Relative inequality	Relative inequality					
Health spending per capita (Contributory health insurance)								
2012	−32.895	2.605	−0.144	47.96	95.08	51.01	30.35	31.37
2013	−23.241	2.449	−0.136	97.81	57.38	25.32	27.54	23.06
2014	−17.283	1.636	− 0.078	64.50	40.89	62.49	29.70	32.18
Health spending per capita (Subsidized health insurance)								
2012	−10.542	1.140	−0.017	89.07	67.69	90.37	68.99	144.51
2013	−13.979	1.220	−0.033	91.80	54.44	79.05	59.71	76.63
2014	−64.781	3.047	−0.168	104.80	76.09	41.93	32.84	134.66
Health spending per capita (Overall: contributory + subsidized scheme)								
2012	−46.275	2.055	−0.114	98.70	79.69	52.38	80.54	53.78
2013	− 73.227	5.003	−0.221	112.89	60.23	53.38	38.03	34.81
2014	−73.052	5.355	−0.227	125.10	65.25	44.87	42.61	36.12

Source: prepared by authors from study results

Secondly, the qualitative data analysis used semi-structured interviews. The recruitment strategy was based on a list of potential participants provided by the Ministry of Health and Social Protection. The eligibility criteria focused on the higher levels of MMR among geographical regions (highest, average, and lowest MMR). The methods of selection of participants was done through email invitation to 43 individuals. Of these, 28 participants responded to the invitation and agreed to be interviewed; 7 participants did not attend the interview. We used data collected from interviews of two different groups of key informants: 8 Health financing decision makers, and 13 individuals who are in charge of maternal policy development and program implementation. Interviews were applied to participants from all three groups of regions until no new themes emerged from the interviews. The saturation point in this study seemed to have been reached at a total of 21 interviews. No financial reward was offered to participants in this research. Multi-panel quantitative data were stored and managed in Epidat, and interviews were transcribed in full and stored and coded into emerging themes using NVivo 10.

### Data analysis and statistical methods

The maternal mortality ratio represents the risk associated with each pregnancy, i.e. the obstetric risk. It is also an SDG indicator. The maternal mortality ratio is the most widely used measure of maternal deaths. The ratio is calculated by dividing recorded (or estimated) maternal deaths by total recorded (or estimated) live births in the same period and multiplying the result by 100,000 [17]. The measurement requires information on pregnancy status, the stage of pregnancy in which death occurred (during pregnancy, during childbirth, or within 42 days of termination of pregnancy), and cause of death. We calculated maternal mortality ratios by contributory, or subsidized health insurance scheme at subnational level as well as a totalized number. A scatter plot was used to describe the distributions of change in maternal mortality ratios according to changes in health spending across regions in Colombia. A scatter plot uses dots to represent values for two different numeric variables and are used to observe the relationship among variables.

### Summary measures of inequality

In order to assess the patterns of inequality in maternal mortality ratios driven by health spending per capita, two summary measures of inequality were calculated using the library for monitoring health inequality in Epidat. First, Slope Index of Inequality (SII) was utilized to explore the gap between worst-off and better-off groups of regions ranked from lowest to highest in health spending per capita. SII revealed the general extent and size of inequality. Second, the Concentration Index (CIX) was used to measure inequality in one variable over the distribution of another. The CIX had a 95% CI with standard error. Higher numerical values indicate more pronounced inequality, whilst lower values indicate smaller inequality. Negative values indicate that more disadvantaged regions (pro-poor distribution) encounter higher inequality, which in this case means regions with lowest health spending per capita.

Absolute measures allowed to assess the progress in reducing inequality, while relative measures allow to determine whether inequalities are being eliminated. SII expresses absolute inequality, whereas the CIX expresses relative inequality [20–22]. These summary measures were disaggregated by health spending per capita quintiles (five groups with the similar number of live births in each), and at three points in time according to the availability of multi-year panel (2012, 2013 and 2014). The analysis by quintiles allowed us to organize the population according to the social determinants that generate inequality [23], which in our case is health care spending per capita, and indicated the gaps that a determinant generates on a health outcome such as maternal mortality. The quintile of health care spending per capita was obtained as we describe as follows:

- ▪ First Step: Rank the panel of data by health care spending per capita.
- ▪ Second Step: Estimate the values by quintiles of health care spending per capita.
- ▪ Third Step: Classify the departments by quintiles. Then, with the value of the quintiles, the departments must be identified into each quintile.
- ▪ Fourth Step: Estimate indicators by quintile. In this step, we required population weights by quintile.
- ▪ Fifth Step: Estimate the population proportion by quintile. Next, it was necessary to calculate the population proportion for each department and for each quintile.
- ▪ Sixth Step: Estimate health care spending per capita and maternal mortality ratio by quintile.

This method is particularly useful in unmasking important geographical inequality, in order to better design targeted interventions, and it is highly recommended by WHO [21, 24].

### In-depth interviews

To understand the key factors of the implementation of health financing reforms and their impact on the maternal health policy implementation, we conducted interviews with key informants within the Colombian health system. Research participants were selected from the Ministry of Health and Social Protection, health services purchasing entities, and health facilities in six selected regions based on maternal mortality ratios (highest, average and lowest levels). Face-to-face interviews with the two different groups of participants were conducted: senior staff in charge of maternal health policy implementation, and health financing decision makers and senior planning officers at the Ministry of Health and Social Protection.

Interviews took between 30 and 60 min and addressed the following topics: (a) universal health coverage (who is covered), (b) health services delivery (which services are covered), and (c) financial protection (what portions of the costs are covered). Each participant signed an informed consent form for each interview. All interviews were conducted in Spanish and all participants agreed to the interview being audio-recorded. Immediately after each interview, observational field notes were taken. Questionnaires for the interviews were translated from English to Spanish. Responses were organized and coded into emerging topics of interest.

### Socio-ethical and gender considerations

Ethical approval was granted by the IDRC’s Advisory Committee on Research Ethics (ACRE) on 20.05.2016. After verification, no additional ethical approval was required in Colombia. We paid special attention to any potential gender or power considerations during the in-depth interviews; no individual was excluded due to their gender. However, we were sure to include as many female respondents as male respondents.

## Results

The results are presented according to the objectives of this study. Firstly, to explore the association between changes in maternal mortality ratios with changes in health spending per capita; secondly, to assess the patterns of inequalities in maternal mortality driven by health spending per capita; and finally, to understand the implementation gaps in health financing reforms which impact maternal health policy implementation.

### Quantitative findings

#### Maternal mortality and health financing

Maternal Mortality Ratios have dropped by a quarter in a 10 a year-period (2005–2014), which means a progress in the reduction of maternal deaths of 23.51 percentage points. In other words, the maternal mortality ratio fell from 70.14 deaths in 2005 to 53.65 per 100,000 live births in 2014, which means the country did not reach the MDG-target. While at the national level mortality decreased, maternal mortality varied significantly at the subnational level over time; there are significant differences between health insurance schemes. It is possible to identify two groups: those regions with relatively higher reductions and those with increases. On the other hand, about a third of territories have reduced their maternal mortality by two thirds during the same period. We found mixed trends when observing data health scheme.

**Fig. 2:**
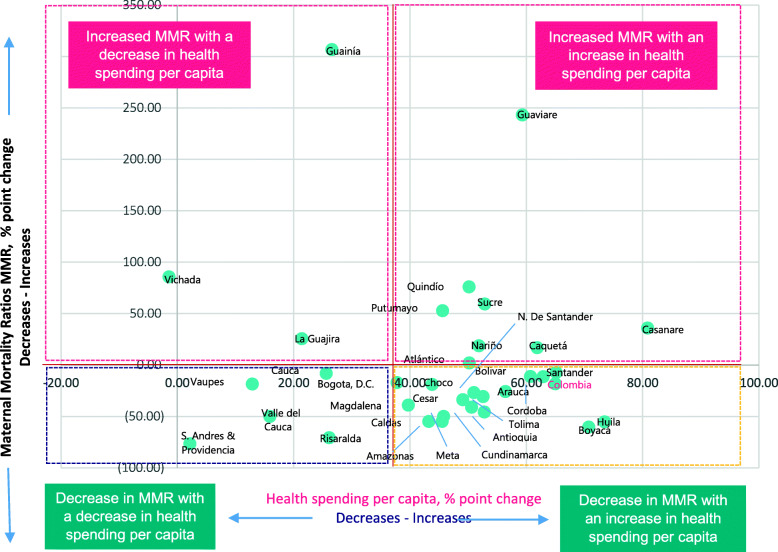
Changes in maternal mortality ratios and health spending per capita at subnational level in Colombia, 2012 and 2014**.** Source: prepared by authors from study results

Figure 2 shows the changes in maternal mortality and health spending per capita at subnational levels in Colombia in percentages point changes. This scatter plot uses dots to represent values for two different numeric variables. The position of each dot on the horizontal and vertical axis indicates values for each territory data point (represented by green dots). Scatter plots are used to observe relationships between variables. The Y-axis scale (from − 100 to 350) represents the changes in Maternal Mortality Ratios (MMR) in percentages points, whilst the X-axis scale represents the percentage point changes in the overall health spending per capita (from cero to 100).

The figure shows four possible associations between the two variables: First, the regions at the top left indicate an increase in maternal mortality, and a decrease in health spending per capita. Second, regions at the top right represent the regions which had an increase in maternal mortality and an increase in health spending per capita. Third, (bottom right) there are regions that had a decrease in maternal mortality and received an increase in health spending per capita. Finally, the regions ate the bottom left had a decrease in maternal mortality with a decrease in health spending per capita. Overall, 50% of the regions are in the bottom right part of the chart, showing a correlation between health spending and reduction in maternal mortality. However, the overall chart shows that this association is not linear, nor does it move in the same direction always.

#### Absolute and relative inequality

Table 1 shows the Slope Index of Inequality, the estimated Concentration Index CIX and the maternal mortality ratios disaggregated data by health spending per capita quintiles at three points in time (2012, 2013 and 2014). To interpret each index, it is important to note that higher numerical values (**) indicate a more pronounced inequality, while lower numerical values indicate lower inequality. It is also worth highlighting that the negative values represent a pro-poor distribution of resources which is associated with the lowest health spending per capita, indicating inequality is affecting the most disadvantaged regions. On the other hand, positive values indicate that inequality impact more advantaged regions (pro-rich distribution), which in this case means regions with highest health spending per capita.

Overall, absolute and relative inequality assessed by both summary measures widened substantially between 2012 and 2014, but mostly due to inequities in the subsidized scheme. In 2012, the magnitude of inequality in the three indicators had a pro-poor inclination. However, the inequality changed between 2012 and 2014, with absolute inequality increasing from − 46.2 to − 73 overall (contributory and subsidized schemes); and from − 10.5 to − 64.7 in the subsidized health insurance scheme while falling from − 32.8 to − 17.2 in the contributory scheme. Similar patterns were observed for the relative inequality, which increased in overall measurements and in the subsidized scheme over that period, going from 2 to 5.3 and 1.1. to 3 respectively. In the contributory scheme, however, numbers went from 2.6 to 1.6.

In 2014, the subsidized scheme shows the largest absolute inequality as evidenced by the SII increase in the overall category — going from − 46.2 to − 73.8 maternal deaths. The growth from the left side indicates that there is a significant difference between the groups in worst-off regions and those in better-off regions. In contrast, maternal deaths in subsidized health insurance increased (which is represented by − 65), whereas in the contributory scheme decreased (from − 32.8 to − 17.2); in other words, relative inequality decreased one point (down from 2.6 to 1.6).

**Fig. 3:**
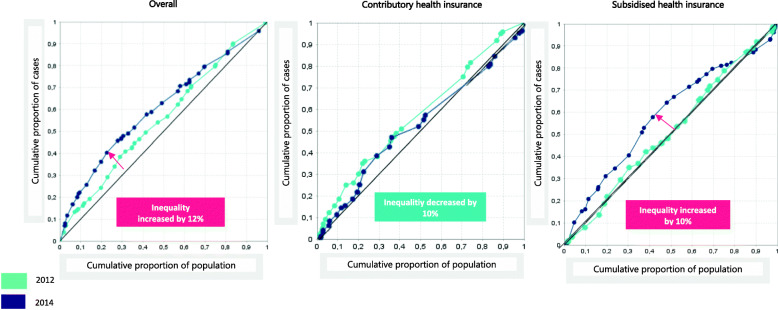
Change of concentration of inequality in maternal mortality by health spending per capita and health insurance schemes in Colombia, 2012 and 2014. Source: prepared by authors from study results

Figure 3 presents the change in concentration of inequality in maternal mortality by health spending per capita and health insurance schemes in Colombia in 2012 and 2014. This plot shows the territories in green and red colored dots to differentiate between the years. Also, shows that the CIX went up from − 0.114 to − 0.227, indicating that in 2012 roughly 25% of all maternal deaths accrued in the territories with the lowest health spending per capita. However, this inequality remained disproportionately concentrated in the same 20% of population and increased by + 12 in the territories with the lowest health spending per capita in 2014. Thus, in 2014 the 20% of disadvantaged regions yielded 36% of maternal deaths.

Closer examination of Inequity by disaggregated quintile revealed that maternal mortality ratios tended to be lowest in quintiles 4 and 5 in the contributory scheme; however, were generally highest in quintiles 1 and 5 in the subsidized scheme and the overall measures, and increased only in quintile 1 over time. Overall, regions reported a wider range of maternal mortality ratios in quintile 1 as compared to quintile 5 in health insurance scheme. In 2012, quintile 3 had higher maternal mortality ratios than quintile 1 in the contributory and subsided schemes. Substantial inequality in maternal mortality was noted in both disadvantaged regions (Q1 and Q2) and the most advantaged regions (Q5). This unusual pattern in the subsidized health insurance scheme is explained as pro-rich and pro-poor inequality: firstly, maternal mortality is higher within the 20% of territories with highest health spending per capita; secondly, maternal mortality is also higher within the 20% of territories with lower health spending per capita.

**Fig. 4:**
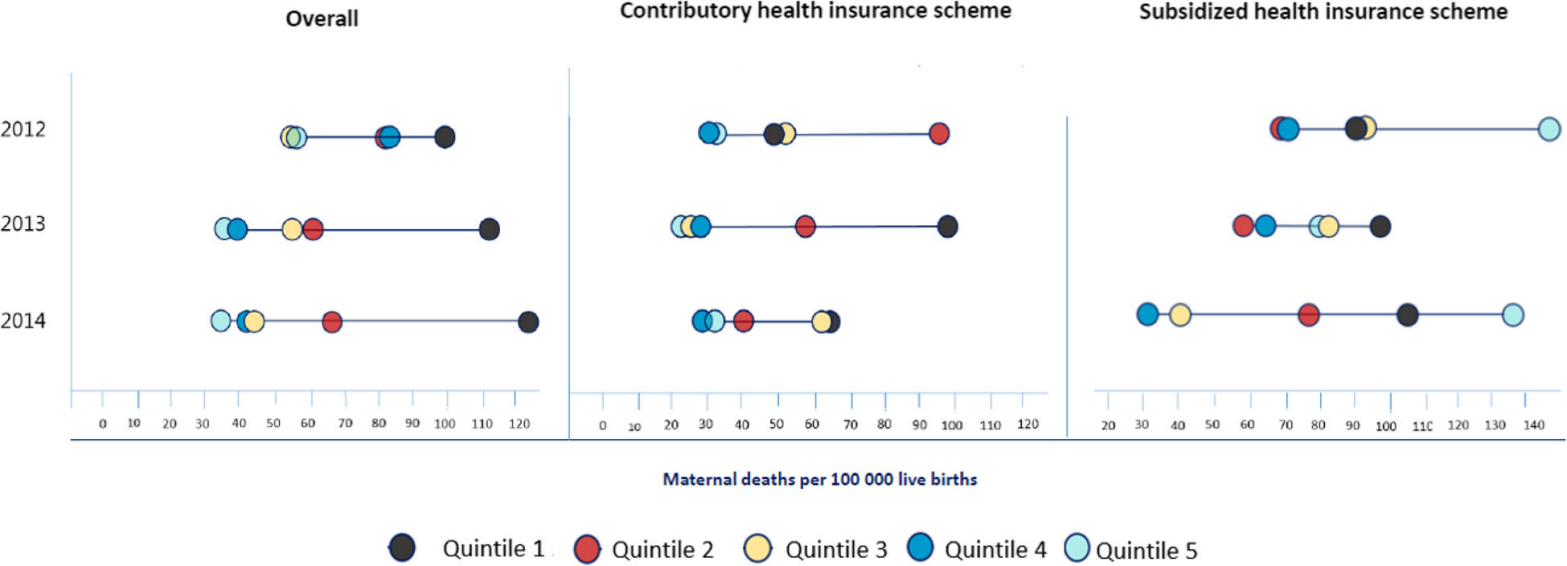
Patterns of inequality in maternal mortality disaggregated by health spending and health insurance scheme in Colombia, 2012–2014. Source: prepared by authors from study results

Figure 4 shows the patterns of inequality in maternal mortality disaggregated by health spending and health insurance scheme in Colombia, 2012–2014. This graph is known as an equiplot and shows the sequence of dots of maternal mortality ratios in a flat line. This Equiplot disaggregated data is presented for health spending per capita quintiles (2012, 2013 and 2014). The farther to the right the sequence of dots are, the higher mortality they represent. Each dot signals one health spending per capita quintile, from the lowest health spending (black and red dots) to the highest health spending (light and dark blue dots). This equiplot presents inequality in maternal mortality in Colombia analyzed by health spending per capita quintiles. Estimates are presented for three comparable years (2012–2014) and by subsidized, contributory, and combined (overall) health insurance scheme.

The dots are connected by a line; longer lines indicate larger absolute inequality and shorter lines represent lower inequality. The results show that health spending related inequality in maternal mortality was smaller in the contributory insurance scheme compared to the subsidized insurance scheme in 2014. Despite the overall decrease, we can see that Q1 showed a particular pattern: increased inequality and mortality, which made 2014 the most inequitable year. In the contributory scheme important inequality persisted for Q4, and remained almost unchanged over time. Simultaneously inequality and mortality have increased in Q1 from 2011 to 2014. Conversely, Q3 and Q4 showed greater reductions. An unusual pattern was observed in Q1, Q2 and Q5 over time: inequality and mortality increased at the same time, which made 2014 the most inequitable year. In the subsidized scheme, absolute and relative inequality increased, especially in the last year.

### Qualitative findings

**Fig. 5:**
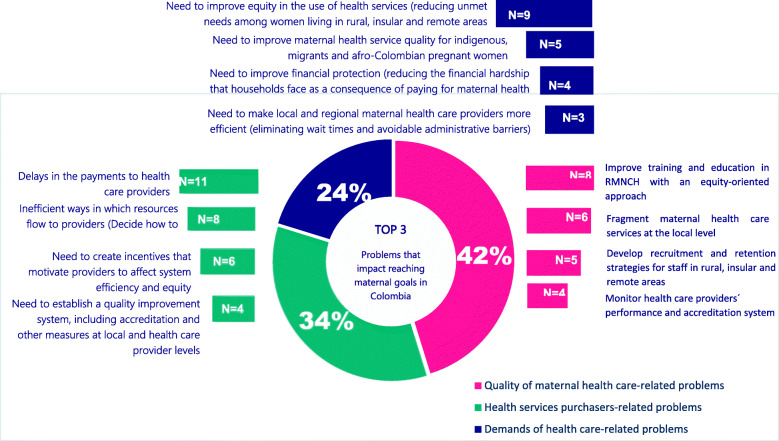
Common problems that impact maternal health coverage and policy implementation in Colombia. Source: prepared by authors from study results

The numbers of individuals that participated in the qualitative study was 21: 14 participants were female and 7 were male. Thirteen senior staff in charge of maternal health policy implementation, and eight health financing decision makers and senior planning officers from the Ministry of Health and Social Protection. The subjects are either working with or at one of six health services purchasing entities, four local health facilities and three local hospitals. Figure 5 shows the common problems that impact maternal health coverage and policy implementation in Colombia. Based on the agreement and disagreement among the health financing policy makers and managers and those in charge of the maternal health policy implementation, three categories emerged during the in-depth interviews: i) problems related to the purchase of health services; ii) problems related to the quality of maternal health care; and iii) problems related to demands made by users.

#### Problems related to the quality of maternal health care

The first theme that emerged in the qualitative analysis is associated with the common problems in the quality of maternal health care that impact the implementation of maternal health policies and achievement of maternal health goals. There was a greater agreement between participants on the needs to improve training and education in equity-oriented Reproductive, Maternal, and Newborn Health (28%); the fragmentation of maternal health care services (26%); the need to develop recruitment and retention strategies for staff in rural, insular and remote areas (23%); the need to monitor performance of health care providers (16%) and problems related with supplies and access to medicines (7%).

Key informants identified the poor medical and nursing training and education programs in Reproductive, Maternal, Newborn and Child Health RMNCH as one of the root causes for lower quality maternal health care. Furthermore, many of the respondents highlighted their concern with the unavailability of health workers due to high rotation, a lack of opportunity and low remuneration. The lack of access to staff is mainly affecting maternal health care delivery in rural, insular and remote areas. The key informants also spoke about the fragmentation of the maternal health care services. They explained that due to the geographical distribution of certain areas, antenatal care, skilled birth attendance and hospitalization care take place in different places and delivery is carried out by another staff, which has affected the continuum of care mainly among the most vulnerable and poorest pregnant women.

#### Problems related to the flow of resources

The second topic that emerged is associated with the common problems that stem from the relationship between purchasers and health care providers within a market of multiple purchasers. The key informants agreed that delays in the flow of the resources (31%) is the most common problem that has considerable impact on maternal health outcomes. They highlighted the issue that many intermediaries were involved in the flow of resources as part of the health financing decision-making. Many participants emphasized the inefficient ways in which resources flow to providers and how they decide to pay: provider payment mechanisms (26%). Several respondents noted that the delays and inefficiency could respond well to incentives that influence the provider’s behavior, thus affecting system efficiency and equity (20%); and to the establishment of a quality improvement system, including accreditation and other measures at local and health care providers (10%); only few research participant agreed on the idea that a limited health financing level exists (5%).

#### Problems related to the demand for health care

Finally, the third main topic that emerged is associated with problems related with the demand for and delivery of health care. Most of the respondents spoke about the need to improve equity in the use of health services (reducing unmet need among women living rural, insular and remote areas (44%). They pointed out the impact of geography and environment in delivering affordable maternal health care. Similarly, they emphasized the need to increase and adapt health care delivery to address ethnic and cultural traditions and taboos. Additionally, they highlighted the many problems regarding the need to improve maternal health service quality to indigenous, migrants and afro-Colombian women who pregnant (23%). These issues that were pointed out are affecting the progressive realization of universal health coverage, mainly among social marginalized and vulnerable pregnant women on the basis of economic status and social class.

## Discussion

Our results identified patterns of socioeconomic and health financing inequality in maternal mortality in Colombia using a mixed research methods approach. During the period analyzed, maternal mortality reductions in most of the departments was not continuously sustained over time; but progress in improving maternal survival accelerated in the 2005–2014 period for the population in the subsidized insurance scheme. Overall, the results suggest a correlation between reductions in maternal mortality ratios and increases in health spending per capita. When maternal mortality ratios declined in most regions, in the considered period, this was associated with an increase in health spending. In fact, a possible explanation for some of the changes may be related to health financing reforms, such as the alignment of the Benefit Package Review for both schemes in 2012, which positively impacted maternal health in 2013. Additionally, the years 2008 and 2013 showed the greatest reductions in maternal mortality in seeming response to the implementation of the National Health Plan and National Strategies to Improve Reproductive, Maternal and Newborn Health (2007) and the 10-year Public Health Plan (2012). There is a different association variation between changes in maternal mortality and changes in health spending per capita, but generally this association was nonlinear, nor did it move in the same direction always. This presents an opportunity for implementing research mainly in the regions in the top right and bottom left of Fig. 2 which present variations in health outcomes in relation to health spending.

The analysis revealed that inequality within the contributory insurance scheme declined, whereas inequality in the subsidized scheme significantly increased, which impacted the overall measurement of inequality. With regard to the disaggregation of data by health spending per capita quintiles, although it is worth noting that maternal mortality ratio has decreased among regions with lowest health spending per capita, high numbers of maternal deaths are still occurring in the regions the lowest resources. Attention should be focused on the 20% of the regions with the lowest health spending per capita wherein maternal mortality has reached 35%.

The models with disaggregated data allowed the identification of common characteristics of inequality in maternal mortality explained by health financing. Three patterns of inequality were found, and each pattern of inequality prompts a different policy response. First, a marginal exclusion pattern –also called top inequality is affecting 60% of regions that receive lower health spending per capita in the contributory scheme. Secondly, social exclusion also called pro-rich inequality was evidenced to impact 20% of regions which despite receiving higher health spending per capita, they still present the worst maternal mortality outcomes. And thirdly, we found mass deprivation in the contributory scheme –also called as bottom inequality, which affects the worst-off group (40% regions with the lowest resources). Nonetheless, such mass deprivation has been reducing over time.

These patterns of inequality have implications for the provision of maternal health care services, mainly where different patterns are seen for regions across different geographical areas. Moreover, a special approach would be required for quintile 4 within the contributory scheme, for which inequality has remained unchanged over time. This might be due to a slower pace of mortality reduction among this group of departments. Where progress is most evident is in better-off regions (quintile 4 and quintile 5 which coincide with main cities and urban geographical areas) whilst the worst-off regions (quintile 1 and quintile 2 which coincide the most rural and remote geographical areas) are left behind. Another explanation is that maternal health policy implementation (such as “Saves Babies”, and “preventable maternal mortality”) in support of maternal health coverage is focusing and reaching only urban areas, instead of addressing the needs of the hard to reach women in rural and remote geographical areas.

We contrasted findings from quantitative data with the topics that emerged from qualitative findings, with the purpose to understand the patterns of inequality in Colombia. These results suggest that the problem goes beyond money and access to resources. Patterns and trends of inequality must also consider the quality of health care providers, as well as the efficiency and transparency of payment mechanisms between healthcare providers and purchasers rather than only geographical and demographic variables. Also, it must be noted that problems related to the improvement of equity-oriented training and education in Reproductive, Maternal, Newborn and Child Health, delays and inefficient ways in which resources flow to providers, and challenges to reduce unmet needs among women living rural, insular and remote areas may be jeopardizing the achievement of upcoming SDG and national goal deadlines. The overall interpretation of this study is that moving forward with maternal health coverage requires addressing health financing issues as part of health policies to improve access to quality and affordable maternal health care, rather than in isolated way.

Our findings contrast with substantial literature that has addressed this issue; prior studies addressed this research question in 89 low-income and middle-income countries [25]; another study in 24 European countries [26]; and others focused on African countries using data from 46 [9] and 47 nations in two periods (1999–2004, and 2000–2013) [27]. Studies have highlighted problems such as the lack of access and inequity in the use of maternal services in Latin American and Caribbean countries [28, 29]; others studies have also shown that effectiveness of health financing policies, in particular highlighting that equity in the allocation of resources was more likely associated with improvement in health outcomes, such as a reduction in maternal mortality [13, 16, 29–32]. To date, studies to understand the health financing inequality in maternal mortality using mixed research methodology and focused on Latin American might be limited.

The strengths of this study includes the use of a multipanel plots with no missing data from 33 different subnational governments, which made it possible to use models to determine whether inequality is being eliminated or to unmask important geographical inequality. There were, however, some limitations to consider: i) the multipanel was not built for a longer period of time due to limitations of different data sources; ii) time and funding constraints also limited the time to complete the data collection during fieldwork; and, iii) there are more dimensions of inequality that represent a source of social exclusion.

## Conclusion

Health financing is a critical component towards improving maternal health. However, beyond the key issues in health financing, issues of quality of care must be addressed. The most promising interventions now could well be those focusing on effective targeted equity-oriented interventions. Putting them in place requires innovation, and different interventions and solutions to address patterns of inequality in maternal mortality driven by health spending per capita. Firstly, afro-Colombian, indigenous, poorer and migrant women must be put at the center of the maternal health care services; this means to address the needs of the hardest women to reach taking into consideration their particular life situations. Secondly, attention should be given to addressing quality of health care through better skills, Reproductive, Maternal, Newborn and Child Health’s training and retention strategies of health workers training in rural, insular and remote geographical areas. This may help to foster socio structural changes and remove common remaining barriers in essential maternal health care services.

Thirdly, the policy response might need to shift, and with it, the efficiency and equity of provider payment mechanisms, the incentives that influence provider’s behavior, and the quality of improvement systems, including accreditation and other measures at local and health care provider levels. A better understanding of payment mechanisms by which health services purchasers and providers interact within the market and through subnational and local different contexts is crucial to make programmatic efforts to reduce inequality in maternal health outcomes.

Finally, given the diversity seen in patterns of health financing-related inequality in maternal mortality, there is a clear and considerable variation in the ways in which different subnational governments are addressing and meeting their goals. A better understanding of these patterns of inequality prompts a call for a targeted approach. In other words, the country must define its own approach to financing for maternal health coverage given its unique context and starting point.

## Data Availability

The datasets supporting the conclusions of this article are available in the Repositorio Institucional Digital RID in https://www.minsalud.gov.co/sites/rid/Lists/BibliotecaDigital/RIDE/VS/ED/GCFI/Copia de RMM_TMIRA_TMEDA_DEPTO_MPIO.zip.
